# Clinical Characteristics and Etiology of Terminal Ileum Ulcers: A Retrospective Study

**DOI:** 10.5152/tjg.2024.23589

**Published:** 2024-08-01

**Authors:** Nomingerel Tseveldorj, Cemre Gündüz, Yaşar Ozan Saracoğlu, Ülkü Dağlı

**Affiliations:** 1 Department of Gastroenterology, Başkent University Faculty of Medicine, Ankara, Türkiye; 2 Department of Internal Medicine, Başkent University Faculty of Medicine, Ankara, Türkiye

**Keywords:** Terminal ileal ulcer, Crohn’s disease, active ileitis

## Abstract

**Background/Aims:**

Terminal ileal ulcers can have various etiologies, including Crohn’s disease (CD), infections, and medication-related causes. This study aims to investigate the incidence of terminal ileal ulcers detected during colonoscopies, explore their underlying causes, and analyze their clinical, endoscopic, and histopathological characteristics. Additionally, the study aims to identify predictive factors that indicate the need for follow-up.

**Materials and Methods:**

Medical records of all patients who underwent colonoscopies, between 2009 and 2019 were retrospectively reviewed. Patients with terminal ileal ulcers, with or without ileocecal valve involvement, were included in the study. Demographic information, medication usage, symptoms, colonoscopy findings, and histopathological data of these patients were analyzed.

**Results:**

A total of 398 patients were included in the study. Histopathological examination revealed that 243 patients (61%) had active ileitis, and 69 patients (17.4%) had chronic active ileitis. The final diagnoses for ulcers were: nonspecific ulcers in 212 patients (53.3%), CD in 66 patients (16.6%), and non-steroidal anti-inflammatory drug-induced ulcers in 58 patients (14.6%). In the multivariate analysis, the parameters predicting CD included the presence of 10 or more ulcers (odds ratio (OR) = 7.305), deep ulcers (OR = 7.431), and edematous surrounding tissue (OR = 5.174), all of which were statistically significant (*P* < .001).

**Conclusion:**

Upon final evaluation, only 66 patients (16.6%) were diagnosed with CD, while 212 patients (53.3%) had nonspecific ulcers. The majority of patients with healed ulcers exhibited pathological findings consistent with active ileitis. Therefore, it can be concluded that not all terminal ileal ulcers are indicative of CD. In those cases with active ileitis, repetitive colonoscopies should be reconsidered.

**Main Points**

- Need for accurate diagnosis: with a rise in detected terminal ileal ulcers through colonoscopy, ensuring definitive diagnoses becomes pivotal. This approach enhances patient care, curtails unnecessary treatment, and prevents over-prescription.
- Comprehensive exploration: we delve into distinct traits, origins, and histopathological attributes of terminal ileal ulcers. Our aim extends to proposing guidelines for identifying patients necessitating follow-up care.
- Non-specific nature: the majority of incidentally found terminal ileum ulcers display non-specific traits, often showing pathology related to active ileitis or other non-specific factors. Implication: terminal ulcers ≠ exclusive sign of Crohn’s disease.
- Medication scrutiny: clinicians should also question medications that can cause terminal ileum ulcers, especially nonsteroidal anti-inflammatory drugs and acetylsalicylic acid. These ulcers can be a cause of overdiagnosis.
- Predictive endoscopic features: a core focus is on uncovering predictive endoscopic traits aiding the diagnosis of terminal ileal ulcers.

## Introduction

Terminal ileitis refers to chronic inflammation of the terminal ileum, characterized by both superficial and deep ulcers that do not involve the ileocecal valve or colon mucosa.^1,2^ The clinical significance of terminal ileitis remains uncertain, particularly in developing countries where both infectious and noninfectious factors contribute to its prevalence.^1^ In recent times, the increased frequency of colonoscopies and terminal ileum intubation has led to a higher detection rate of terminal ileum lesions, fostering curiosity and awareness about this condition.

Terminal ileitis can be encountered incidentally in asymptomatic patients or can manifest with symptoms such as acute right lower abdominal pain, fever, or diarrhea. In cases of acute infectious ileitis, bacterial or viral infections like cytomegalovirus (CMV), salmonella, and yersinia are common culprits.^1^

Various factors can give rise to terminal ileum ulcers, encompassing conditions like Crohn’s disease (CD), tuberculosis, Behcet’s disease, infections, malignancies, and the use of nonsteroidal anti-inflammatory drugs (NSAIDs).^1,3,4^ Crohn’s disease is characterized by chronic and recurring symptoms, often leading to complications such as obstruction, hemorrhage, fistulization, and extraintestinal manifestations. The ileum or ileocolonic mucosa is affected in 60%-70% of CD patients.^4^

Detecting terminal ileitis presents a clinical challenge. A misdiagnosis of CD could result in unnecessary and potentially harmful long-term treatments, while diagnosing intestinal tuberculosis exposes patients to the risks associated with antituberculosis therapy.^1^

Differentiating between terminal ileum ulcers that require further evaluation and those that can be managed symptomatically is crucial. Our study aims to analyze the incidence, causes, and clinical, endoscopic, and histopathological features of terminal ileum ulcers discovered during colonoscopies conducted over the past decade for various reasons.

## Materials and Methods

### Data Collection

We obtained data from an online hospital medical records database containing records of all colonoscopies conducted at Başkent University Hospital between January 2009 and October 2019. Patients with terminal ileum ulcers, including those with ileocecal valve ulcers, were selected as the focus of our study.

### Inclusion and Exclusion Criteria

Our inclusion criteria encompassed patients aged 18 years and older with documented terminal ileum ulcers detected during colonoscopies. Exclusion criteria encompassed individuals under 18 years of age and those with a pre-existing history of inflammatory bowel disease, Behçet’s disease, tuberculosis, or malignancy. Patients with colonic ulcers were also excluded from our study.

### Data Collection and Analysis

We meticulously reviewed and documented various aspects of the selected patients’ profiles, including demographic information, medication use (such as aspirin or NSAIDs), medical history, clinical presentation, and findings from colonoscopic examinations. All patients underwent biopsies, and corresponding histopathological findings were recorded. Additionally, we assessed the patients’ diagnoses, clinical progress, and management strategies to ascertain any changes following subsequent colonoscopic evaluations. Although specific follow-up data confirming patient diagnoses were not formally recorded, we conducted thorough cross-referencing.

The primary outcome of our study is the etiology of incidental terminal ileum ulcers. The secondary outcomes include the relationship between etiologies and histopathology, colonoscopic features, and the outcomes of terminal ileum ulcers.

### Diagnostic Procedures

In cases where clinical and ulcer characteristics indicated potential tuberculosis or CMV infection, we employed the polymerase chain reaction method for diagnosis. To confirm the diagnosis of CD, we also needed to perform magnetic resonance)/computerized tomography enterography and fecal calprotectin tests.

### Ethics Committee Approval

Written informed consent was obtained from all patients before undergoing the procedures. The study protocol was approved by the Başkent University Institutional Review Board (Project No: KA21/140, date: March 23, 2021).

### Statistical Analysis

We performed statistical analyses using the Statistical Package for the Social Sciences 25.0 for Windows. Descriptive statistics, including mean ± SD for continuous variables, and frequency (percentage) for discrete variables, were calculated in line with the study’s context. Categorical variables were subjected to analysis using the chi-square and Fisher’s exact test. To explore potential risk factors, we initially conducted univariate analyses to identify relevant variables. Subsequently, a multivariate logistic regression model was constructed for more comprehensive analysis. A significance level of *P* < .05 was considered statistically significant.

## Results

A total of 21 010 colonoscopic procedures were conducted in our gastroenterology unit over a span of 10 years. After applying exclusion criteria, including patients without intubation of the ileum due to various reasons, those with colonic ulcers, and those below 18 years of age, a cohort of 398 patients with terminal ileal ulcers, either with or without ileocecal valve involvement, were included in our study. Of these patients, 203 (51%) were male, 195 (49%) were female, and the mean age was 53 years (ranging from 23-92 years). Approximately, 49.8% of patients had no chronic diseases, with essential hypertension (20.9%) and rheumatologic diseases (9.8%) such as ankylosing spondylitis and familial Mediterranean fever being the most common chronic conditions. A history of acetylsalicylic acid (ASA) usage was reported by 59 (14.8%) patients, and 86 (21.6%) had a history of NSAID usage.

### Clinical Presentation

The most frequent clinical presentations were abdominal pain (39.9%), diarrhea (20.6%), and overt gastrointestinal bleeding (OGIB) (17.1%). A majority of patients (49.5%) reported symptoms lasting 0-6 months (Table 1).

### Colonoscopic Findings

Among the colonoscopies, 351 (88.2%) patients had terminal ileal ulcers only, while 47 (11.8%) had ulcers in both the terminal ileum and ileocecal valve. The observed ulcers included superficial ulcers (79.6%) and deep ulcers (20.4%). The shape of ulcers varied, with aphthous ulcers (47.2%), round ulcers (25.1%), linear ulcers (17.1%), and star-shaped ulcers (10.6%) being detected. Most commonly, the number of ulcers observed was 0-4 (40.5%), and the size of ulcers ranged from 1-4 mm (70.1%) (Table 2).

### Histopathological Findings

Histopathological examination revealed active ileitis in 243 (61%) patients and chronic active ileitis in 69 (17.4%) patients at the first colonoscopy (Table 3).

### Diagnoses and Follow-up

**Figure 1.**
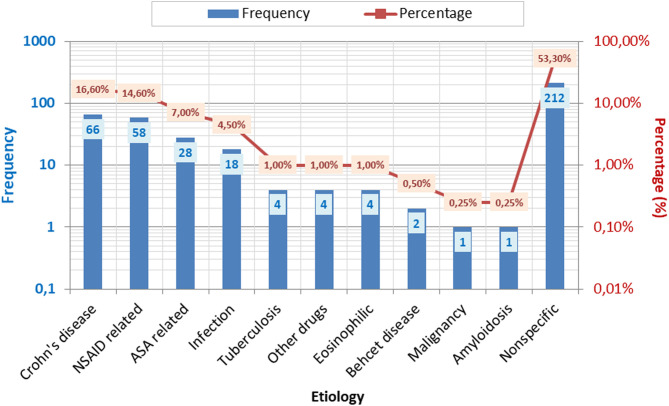
Etiologies of patients with terminal ileum ulcers.

The final diagnoses were as follows: 66 patients (16.6%) were diagnosed with CD, 58 (14.6%) patients had NSAID-induced ulcer, 28 (7%) had ASA-induced ulcer, 18 (4.5%) had infection-related ulcer, 4 patients (1%) had intestinal tuberculosis, and 212 patients (53.3%) had nonspecific ulcers (Figure 1). Out of the patients, 150 (37.7%) came for follow-up examinations, with a median follow-up duration of 35.1 ± 26.7 months. We performed a repeat colonoscopy on only 112 (28.1%) patients who gave their consent and found that 70 (62.5%) patients with terminal ileum ulcers were healed. Control colonoscopy was performed on 112 patients who had suspected unresolved terminal ileum ulcers after treatment, to confirm the diagnosis, or for some patients who had persistent complaints.

**Figure 2.**
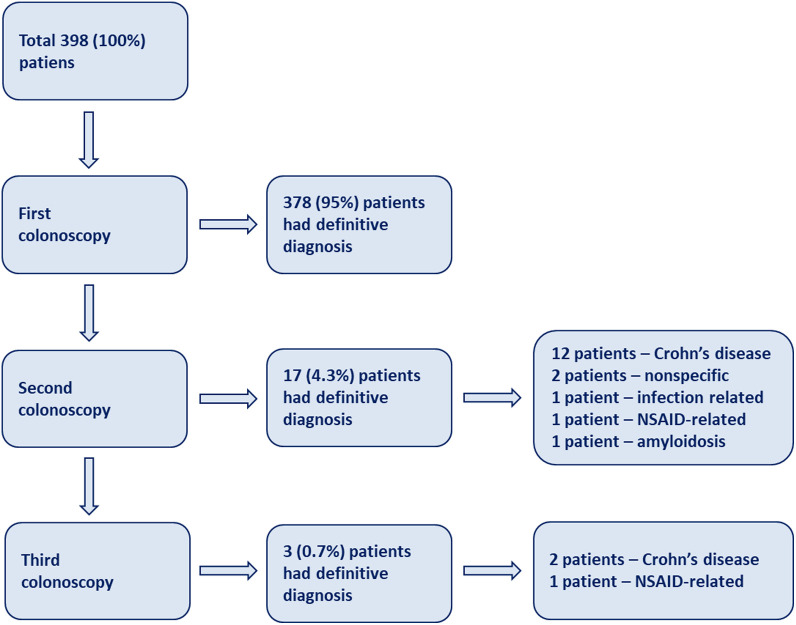
Flowchart of colonoscopies to confirm the definitive diagnosis.

Definitive diagnosis refers to the final diagnosis resulting from all evaluations, which will be followed by this diagnosis, whether it is a nonspecific ulcer or other diagnoses. Notably, most patients (378; 95%) received definitive diagnoses at their initial colonoscopy. The remaining patients needed further colonoscopies to confirm the diagnosis. Therefore, 17 (4.3%) patients required a second colonoscopy, and 3 (0.7%) patients had a third colonoscopy. In the second colonoscopy, a total of 12 patients were diagnosed with CD, and 2 patients were diagnosed with nonspecific ulcers. In the third colonoscopy, 2 patients were diagnosed with CD, and 1 patient with an NSAID-related ulcer (Figure 2).

Median time to diagnosis defines the period until a definitive diagnosis is made in patients after the first colonoscopy. Our median time to diagnosis was 21.7 months. The delay in diagnosis averaged 18.5 ± 13.8 months for the second colonoscopy and 40 ± 9.2 months for the third colonoscopy.

### Predictive Factors and Analysis

In the univariate analysis, CD predicting parameters; 10 and more ulcers (odds ratio (OR) = 9.47), ≥ 10 mm ulcer size (OR = 9.333), linear ulcers (OR = 11.718), deep ulcers (OR = 13.147), and edematous surrounding tissue (OR = 9.231) were statistically significant (*P* < .001) and had a prominently increased risk of having CD. However, in the multivariate analysis of these patients, only the number of ulcers ≥10, the presence of deep ulcers, and surrounding tissue edema were statistically significant (Table 4).

While there was no significant parameter in the univariate analysis of NSAID-induced ulcers, it was observed that the round ulcer had increased the risk in the multivariate analysis (OR = 2.271; 95% CI, 1.042-4.949; *P* = .039). For infection-related ulcers, edema of the surrounding tissue was significant in both univariate and multivariate analysis (OR = 4.917; 95% CI, 1.048-23.057; *P* = .043). There were no statistically significant parameters in univariate and multivariate analysis for nonspecific ulcers. The ulcer size of 5-9 mm (OR = 2.616; 95% CI, 1.153-5.938; *P* = .021) and star-shaped ulcer (OR = 3.56; 95% CI, 1.269-9.988; *P* = .016) were statistically significant in the univariate analysis in ASA-related ulcers. On the other hand, only the ulcer size of 5-9 mm was statistically significant in the multivariate analysis (OR = 3.164; 95% CI, 0.969-10.330; *P* = .056) (Table 5).

### Healing and Outcome

According to the outcomes of re-colonoscopy in our study, among a total of 112 patients who underwent re-colonoscopy, 70 patients (62.5%) demonstrated ulcer healing. Forty patients (35.8% of those who underwent repeat colonoscopy) achieved healing without specific treatment. Of the remaining patients, 18 (16%) received treatment for CD, 1 (0.9%) received treatment for Behçet’s disease, and 11 (9.8%) patients received antibiotic treatment. Among the healed ulcers, in the order of frequency, the most common were nonspecific ulcers in 32 (45.7%), CD in 16 (22.9%), and infection-related ulcers in 7 (10%). Additionally, the majority of healed ulcers were only in the terminal ileum, had ulcer sizes of 1-4mm, and were superficial in nature (Table 6).

In terms of pathology, we performed control colonoscopy in 70 (62.5%) patients with active ileitis and 29 (25.9%) patients with active chronic ileitis. Among these patients, ulcers healed in 44 (62.9% of 70 patients) and 15 (51.7% of 29 patients) patients, respectively.

The histopathological results of all ulcers and healing ulcers on control colonoscopy are given in Table 3.

## Discussion

The presented study contributes valuable insights into the features, etiology, and clinical outcomes of terminal ileum ulcers detected incidentally during ileocolonoscopy. While several studies have explored similar aspects, many of these studies have been conducted in Asian countries, potentially leading to a biased representation of the situation in Turkey, where the prevalence of inflammatory bowel disease (IBD), especially CD, is different.^1,3,5-7^ Similar to Europe, where the prevalence of IBD, especially CD, is 7.7/100 000,^8 ^our study aimed to understand the features, etiology, and clinical outcomes of incidentally detected terminal ileum ulcers on ileocolonoscopy, regardless of symptoms, in a single medical center.

In this study, we investigated data from 398 patients with terminal ileum ulcers, and specific etiologies could be identified in nearly half of the patients (188; 47.2%), providing a broader perspective on the causes of terminal ileum ulcers. We found 66 (16.6%) patients had CD. In a study conducted in Turkey, they found that among 62 patients (6%) with terminal ileum ulcers, 22 (35.4%) had clinically significant histopathological findings, and 12 patients (19.3%) were diagnosed with CD.^9^ In contrast, our study identified a higher number of ulcers with specific etiologies (46.7%), while the percentage of patients with CD was similar (16.6%). Variation in results exists across different studies; for example, a study from India reported a CD prevalence of 25.7%,^5^ whereas a study from Nepal indicated 8.3%.^2^ Additionally, in a study conducted in China, 11.56% of 1099 patients with terminal ileum ulcers were diagnosed with CD.^3^

In our study, the most frequent clinical presentations were abdominal pain (39.9%), diarrhea (20.6%), and OGIB (17.1%). Surprisingly, in another study from China, among 209 patients with isolated small bowel ulcers (ISBU), the most frequent clinical symptom was abdominal pain (54.1%) followed by OGIB (30%). In terms of etiology, CD (106 patients; 50.7%) was the main cause of ISBU. Site stratification by etiology showed that ileal ulcers were more common with CD (57,5%).^10^ The high frequency of clinical symptoms (abdominal pain, OGIB) and CD diagnosis in this study were thought to be because the study included all small intestine ulcers, not just the terminal ileum.

Histopathologic findings of terminal ileum ulcers in our study were predominantly consistent with active ileitis and active chronic ileitis (312; 78.4%). These findings were lower than those of a study from Korea, where 92.7% of 134 patients with terminal ileum ulcers exhibited active and/or chronic ileitis.^5^

Regarding our study’s re-colonoscopy outcomes, among a total of 112 patients who underwent re-colonoscopy, 70 patients (62.5%) demonstrated ulcer healing. Notably, 40 patients (35.8% of those who underwent repeat colonoscopy) achieved healing without specific treatment. This proportion appears lower than reported in other studies. For instance, a retrospective study involving 93 asymptomatic patients with isolated terminal ileum ulcers found that 62 (66.7%) showed ulcer healing on follow-up colonoscopy.^6^ Similarly, incidental terminal ileum ulcers in 97 patients (72.4%) showed improvement on the second colonoscopy.^5^ The inclusion of patients regardless of symptoms, as well as those with a history of ASA and NSAID use (medications occasionally associated with small bowel ulcers,^11,12^ in our study could account for the lower healing rate observed.

In terms of the etiology of healed ulcers, out of 112 patients, 32 (26.6%) had nonspecific ulcers, which accounted for 21.3% among the 150 patients who attended follow-up appointments. Additionally, 16 patients (14.3% of 112 patients) had ulcers related to CD, 10 patients (8.9% of 112 patients) had ulcers related to NSAID/ASA usage, and 7 patients (6.25% of 112 patients) had ulcers related to infection.

As anticipated, among the 70 healed ulcers observed during follow-up colonoscopy, the majority (32; 45.7%) were nonspecific ulcers. Previous studies have also indicated high healing rates for nonspecific ulcers. For instance, 1 study found that 88.9% of patients with nonspecific ulcers responded to symptomatic management, with 84% achieving endoscopic resolution upon follow-up.^7^ Similar findings were observed in a Chinese study where 7 patients were monitored for 7 years.^13^ Another study involving 142 patients with asymptomatic isolated terminal ileum ulcers (ITIU) reported that 60 (64.5%) patients showed complete ulcer resolution without treatment, with 96.8% (90 out of 93) of ITIU patients experiencing a favorable clinical course without adverse event.^6^ These observations support the benign nature of nonspecific terminal ileum ulcers, suggesting a lack of serious complications.

Factors predicting etiologies of incidental terminal ileum ulcers have been insufficiently studied. We hypothesized that endoscopic ulcer features, including shape, depth, number, size, and surrounding tissue inflammation, could predict ulcer etiology. Our study revealed that findings of 10 or more ulcers, deep ulcers, and edema of surrounding mucosa were statistically significant predictors of CD in multivariate analysis. While linear ulcers had the highest OR value (OR: 1.599, 95% CI: 0.492-5.192), the *P*-value was insignificant (*P* = .435). Surprisingly, round-shaped ulcers (*P* = .039) in NSAID-related ulcers and star-shaped ulcers (*P* = .016) in ASA-related ulcers were statistically significant, respectively, in multivariate and univariate analysis.

In a previous study conducted in India, among 45 symptomatic patients with terminal ileum ulcers, 31 were diagnosed with ITB or CD. The presence of deep ulcers on colonoscopy was statistically significant (*P* = .004) when comparing patients treated with specific treatment to those receiving symptomatic treatment.^1^

In our study, despite 14.8% and 21.6% of patients having a history of ASA and NSAID usage, respectively, the etiology of terminal ileal ulcers was found to be only 7% (28 patients) ASA-related and 14.6% (58 patients) NSAID-related. This highlights the importance of laboratory, radiological, and pathological examinations during ulcer diagnosis.

The most common histopathologic finding of terminal ileum ulcers in our study was active ileitis, accounting for 61% of cases. The majority of patients with healed ulcers exhibited pathologic findings consistent with active ileitis (44; 62.9% in re-colonoscopy patients). Based on our data, most terminal ileal ulcers presented with active ileitis, and the majority of them are healed without complication.

The limitations of our study include its retrospective nature, which may have led to insufficient drug questioning, particularly regarding NSAIDs and ASAs. As a result, some cases diagnosed as nonspecific ulcers could potentially have been caused by NSAID or ASA use. Additionally, the loss of some patients during follow-up could have impacted the results, which might differ slightly in a prospective study. However, all colonoscopic findings were recorded based on the definitions provided by the specialist doctors.

Although nearly half of the patients in our study had specific etiologies, the other half presented with nonspecific ulcers. Since endoscopic findings alone cannot reliably predict etiology, laboratory, histological, and radiological examinations are essential. In conclusion, not all terminal ulcers are indicative of CD. Considering clinical presentation, repetitive colonoscopies should be avoided in cases where histopathological evaluation is consistent with active ileitis.

Terminal ileum ulcers can have various etiologies, and not all ulcers are indicative of CD. Clinical presentation, histopathological evaluation, and repetitive colonoscopies are essential to reach a definitive diagnosis and provide appropriate management. Nonspecific terminal ileum ulcers often have a benign course and may not require specific treatment. Further research and follow-up studies are necessary to better understand the clinical significance and optimal management of terminal ileum ulcers.

## Figures and Tables

**Table 1.** Clinical Characteristics of Patients with Terminal Ileum Ulcers

	Number of Patients	Percentage (%)
Gender		
	Male	203	51
Female	195	49
Age	53.2 ± 13.5	
Clinical presentation*		
Abdominal pain	159	39.9
Diarrhea	82	20.6
Overt gastrointestinal bleeding	68	17.1
Anemia	54	13.6
Constipation	52	13.1
Weight loss	22	5.5
Asymptomatic	51	12.8
Comorbidity*		
No comorbidity	198	49.8
Hypertension	83	20.9
Rheumatic disease	39	9.8
Coronary arterial disease	28	7
Malignancy	36	9.1
Type 2 diabetes mellitus	30	7.6
Other disease	30	7.6
Transplantation	11	2.7
ASA usage	59	14.8
NSAID usage	86	21.6
Duration of symptoms		
0-6 months	197	49.5
6 months-1 year	82	20.6
≥1 year	119	29.9

ASA, acetylsalicylic acid; NSAID, nonsteroidal anti-inflammatory drugs.

*Some patients presented more than one symptoms and comorbid diseases.

**Table 2.** Endoscopic Findings of Patients with Terminal Ileum Ulcers

	Number of Patients	Percentage of Patients (%)
Involvement of area		
	Terminal ileum	351	88.2
Terminal ileum and Ileocecal valve	47	11.8
Number of ulcers		
	0-4	161	40.5
	5-9	77	19.3
10 and over	160	40.2
Size of ulcers		
	1-4 mm	279	70.1
	5-9 mm	85	21.4
10 mm and over	34	8.5
Deepness of ulcers		
	Superficial	317	79.6
Deep	81	20.4
Shape of ulcers		
	Aphthous	188	47.2
	Round shaped	100	25.1
	Star shaped	42	10.6
Linear shaped	68	17.1
Ulcer surrounding tissue		
	Non-edematous	148	37.2
Edematous	250	62.8

**Table 3.** Ulcer Histopathologies of Patients in the First and Control Colonoscopies

Pathology	First Colonoscopy (n = 398)	All Re-colonoscopy Patients (n = 112)	Among Healed Patients (n = 70)
Active chronic ileitis	69 (17.4%)	29 (25.9%)	15 (21.4%)
Active ileitis	243 (61%)	70 (62.5%)	44 (62.9%)
Active Peyer plaque	27 (6.8%)	5 (4.5%)	4 (5.7%)
Edematous mucosa	59 (14.8%)	8 (7.1%)	7 (10%)
Total	398 (100%)	112 (100%)	70 (100%)

**Table 4.** Univariate and Multivariate Analysis of Endoscopic Findings of Crohn’s Disease

	Variables	n = 66	Univariate OR	95% CI	Univariate *P*	Multivariate OR	95% CI	Multivariate *P*
Number of ulcers	0-4	8 (12.1%)	–	–	–	–	–	ref
	≥5-9	5 (7.6%)	1.328	0.420-4.203	<.001	2.037	0.571-7.265	.273
	≥10	53 (80.3%)	9.473	4.328- 20.735	<.001	7.305	2.929- 18.219	<.001
Size of ulcer	1-4mm	27 (40.9%)	–	–	–	–	–	ref
	≥5-9mm	22 (33.3%)	3.259	1.741-6.101	<.001	1.304	0.479-3.551	.604
	≥10mm	17 (25.8%)	9.333	4.276- 20.734	<.001	0.805	0.258-2.858	.859
Depth of ulcer	Superficial	24 (36.4%)	–	–	–	–	–	ref
	Deep	42 (63.6%)	13.147	7.197- 24.019	<.001	7.431	2.928- 18.863	<.001
Shape of ulcer	Aphthous	14 (21.2%)	–	–	–	–	–	Ref
	Round shaped	11 (16.7%)	1.536	0.670-3.523	.311	0.228	0.068-0.767	.017
	Star shaped	8 (12.1%)	2.924	1.139-7.510	.026	0.721	0.203-2.562	.613
	Linear	33 (50%)	11.718	5.687- 24.145	<.001	1.599	0.492-5.192	.435
Inflammation of surrounding tissue	No edema	5 (7.6%)	–	–	–	–	–	ref
	With edema	61 (92.4%)	9.231	3.616– 23.566	<.001	5.174	1.784–15.010	.002

OR, odds ratio.

**Table 5.** Univariate and Multivariate Analysis of Endoscopic Findings of Nonsteroidal Anti-inflammatory Drugs, Infection, and ASA-Related Terminal Ileum Ulcers

	NSAID-Related					Infection-Related					ASA-Related				
	N = 58	Univariate *P*	Multivariate OR	95% CI	Multivariate *P*	N = 18	Univariate *P*	Multivariate OR	95% CI	Multivariate *P*	N = 28	Univariate *P*	Multivariate OR	95% CI	Multivariate *P*
Number of ulcer															
0-4	21 (36.2%)	–	–	–	Ref	5 (27.8%)	–	–	–	Ref	13 (46.4%)	–	–	–	Ref
≥5-9	12 (20.7%)	.596	1.294	0.591- 2.835	.519	4 (22.2%)	.434	1.597	0.403- 6.325	.505	7 (25%)	.792	1.287	0.474-3.496	.621
≥10	25 (43.1%)	.510	1.350	0.684- 2.665	.388	9 (50%)	.276	1.604	0.498- 5.170	.429	8 (28.6%)	.270	0.670	0.251-1.788	.424
Size of ulcer															
1-4 mm	41 (70.7%)	–	–	–	Ref	11 (61.1%)	–	–	–	Ref	15 (53.6%)	–	–	–	Ref
	≥5-9 mm	11 (19%)	0.686	0.813	0.479- 3.551	.643	5 (27.8%)	.448	1.188	0.309- 4.573	.802	11 (39.3%)	0.021	3.164	0.969- 10.330	.056
	≥ 10 mm	6 (10.3%)	0.650	1.359	0.258- 2.858	.631	2 (11.1%)	.595	1.354	0.188- 9.736	.764	2 (7.1%)	0.902	2.333	0.328- 16.602	.398
Depth of ulcer															
Superficial	46 (79,3%)	–	–	–	Ref	14 (77.8%)	–	–	–	Ref	24 (85.7%)	–	–	–	Ref
	Deep	12 (20.7%)	0.945	1.046	0.411-2.658	.925	4 (22.2%)	.840	0.671	0.163-2.756	.617	4 (14.3%)	0.412	0.547	0.143- 2.095	.379
Shape of ulcer															
Aphthous	24 (41.4%)	–	–	–	Ref	6 (33.3%)	–	–	–	Ref	10 (35.7%)	–	–	–	Ref
	Round shaped	19 (32.8%)	0.160	2.271	1.042- 4.949	.039	6 (33.3%)	.264	1.290	0.335- 4.970	.711	7 (25%)	0.566	1.287	0.376- 4.413	.688
	Star shaped	8 (13.8%)	0.291	1.995	0.707- 5.626	.192	4 (22.2%)	.083	2.145	0.433- 10.628	.350	42 (10.6%)	0.016	2.853	0.724- 11.249	.134
	Linear	7 (12.1%)	0.593	1.117	0.350- 3.565	.852	2 (11.1%)	.919	0.538	0.075- 3.876	.538	4 (14.3%)	0.861	1.151	0.226- 5.857	.865
Inflammation of surrounding tissue															
No edema	28 (48.3%)	–	–	–	Ref	2 (11.1%)	–	–	–	Ref	14 (50%)	–	–	–	Ref
	With edema	30 (51.7%)	0.061	0.441	0.228-0.855	.015	16 (88.9%)	.034	4.917	1.048-23.057	.043	14 (50%)	0.150	0.431	0.176- 1.059	.067

CI, confidence interval; NSAID, nonsteroidal anti-inflammatory drugs; OR, odds ratio.

**Table 6.** Etiologies and Endoscopic Findings of Patients with Healed Ulcers in the Control Colonoscopy

	Number of Patients	%
Etiology		
	Crohn disease	16	22.9
	Infection	7	10
	NSAID related	6	8.6
	ASA related	4	5.7
	Behcet’s disease	1	1.4
	Eosinophilic	1	1.4
Nonspecific	32	45.7
Involvement of area		
Terminal ileum	57	81.4
	Terminal ileum and ileocecal valve	13	18.6
Number of ulcers		
0-4	27	38.6
	5-9	13	18.6
	10 and over	30	42.8
Size of ulcers		
1-4 mm	48	68.6
	5-9 mm	15	21.4
	10 mm and over	7	10
Deepness of ulcers		
Superficial	51	72.9
	Deep	19	27.1
Shape of ulcers		
Aphthous	33	47.1
	Round shaped	22	31.4
	Star shaped	9	12.9
	Linear shaped	6	8.6
Ulcer surrounding tissue		
Non-edematous	25	35.7
	Edematous	45	64.3

ASA, acetylsalicylic acid.
